# Trajectories in physical functioning at older age in relation to childhood and adulthood SES and social mobility: a population-based cohort study

**DOI:** 10.3389/fpubh.2023.1228920

**Published:** 2023-09-07

**Authors:** Andrzej Pająk, Maciej Polak, Magdalena Kozela, Agnieszka Doryńska, Martin Bobak

**Affiliations:** ^1^Department of Epidemiology and Population Studies, Faculty of Health Sciences, Institute of Public Health, Jagiellonian University Medical College, Krakow, Poland; ^2^Department of Epidemiology, Cardiovascular Disease Prevention and Health Promotion, National Institute of Cardiology, Warsaw, Poland; ^3^Research Centre for Toxic Compounds in the Environment (RECETOX), Masaryk University, Brno, Czechia; ^4^Research Department of Epidemiology and Public Health, University College London, London, United Kingdom

**Keywords:** physical functioning, socioeconomic status, social mobility, aging, cohort study

## Abstract

**Introduction:**

Older age is associated with the deterioration of physical functioning (PF), and low PF is strongly related to poor quality of life among older people. We conducted a study to examine the trajectories of PF between middle and old age, considering sex differences as well as the association between socioeconomic status (SES) at different life stages and changes in PF.

**Methods:**

We analyzed data from the Polish arm of the HAPIEE (Health, Alcohol and Psychosocial factors In Eastern Europe) study, including 1,116 men and 1,178 women aged 45–64 years at baseline. Adult and childhood SES and social mobility were assessed using a retrospectively focused questionnaire. PF was assessed using the 10-question SF-36 scale at baseline examination, face-to-face re-examination, and three postal surveys, covering up to 20 years (on average, 18 years). We employed Generalized Estimating Equations models to assess changes in PF scores over time and compare PF trajectories across different SES categories.

**Results:**

After adjusting for age and other covariates, we found that, in both sexes, participants with always middle or high SES, as well as those who reported upward mobility, had higher PF scores at baseline compared to those with always low SES. A decline in PF between middle and old age was observed in all SES groups; however, the decline was slower in participants with always middle or high SES compared to those with always low SES.

**Conclusion:**

This cohort study revealed that lower SES and downward social mobility were cross-sectionally associated with poorer PF, while upward social mobility seemed to largely reverse the effect of low childhood SES. In addition to the cross-sectional associations observed at baseline, advantaged SES was also significantly associated with a slower decline in PF over an 18-year follow-up period.

## 1. Introduction

Old age is associated with deterioration of physical functioning (PF), which can be attributed to physiological changes associated with aging, chronic diseases, and long-lasting complications from acute diseases. The development of PF limitations is a lifelong process, and it is widely recognized that low PF is strongly related to poor quality of life among older people. Maintaining PF is a key component of healthy aging (1, 2). However, the extent to which age-related decline in PF can be modified by psychosocial, economic, environmental, behavioral, and medical care factors remains uncertain (3).

Socioeconomic status (SES) has been extensively studied as a determinant of health, both within and between countries (4–6). The effect of socioeconomic factors on the health of older people reflects the accumulation of disadvantages throughout their life course (the cumulative advantage/disadvantage hypothesis) (7–11). However, some studies have proposed that social differences in health may decrease in old age (12–14), possibly due to the fact that individuals with poorer health and lower SES are more likely to die before reaching old age (the age-as-leveler hypothesis) (15, 16). Recent analyses of data from the Survey of Health, Aging, and Retirement in Europe (SHARE) have indicated that both social causation and health selection may contribute to social inequalities in health (17).

Individual SES is a complex concept, and the significance of its components varies depending on the culture and overall socioeconomic position of the population (18). In addition to factors such as educational attainment, occupation, and material conditions, childhood circumstances are key determinants of future health. One proposed explanation for social inequalities in health is that early-life disadvantages accelerate biological aging processes (19, 20). Childhood SES has been found to influence health through material, structural, psychosocial, and behavioral factors (21). It has been associated with both physical health and psychological well-being (17, 22).

On the other hand, low SES in adulthood was related to higher exposure to cardiovascular disease risk factors, such as smoking, physical inactivity, overweight, poor dietary quality (23), and unfavorable blood lipid profiles (24). It has also been associated with other general health parameters, including low hemoglobin levels (25) and poor self-rated health (21).

A wealth of evidence exists on the relationship between SES and PF. For example, a recent study of over 5,000 participants in the English Longitudinal Study of Aging revealed that lower SES was associated with greater declines in grip strength, gait speed, chair stand failure, sensory function, cognitive function, depressive symptoms, reduced enjoyment of life, poorer social functioning, and some markers of physiological function (e.g., C-reactive protein, fibrinogen, and lung function), underscoring the critical role of SES in the aging process (26).

Although the relationship between socioeconomic status and physical functioning is well documented, most studies use one point estimate on physical functioning and few address the problem of social mobility. Recently, there has been an increased focus on the life course approach in studying the effects of SES on health. This approach allows for investigations beyond simple measures of childhood and adulthood SES and provides a better understanding of the role of SES exposure in disease development (27, 28). Vertical social mobility, which refers to upward or downward changes in a person’s SES over their life course (29), appears to be particularly important. However, limited evidence is available regarding its effect on aging, specifically on PF in old age. Most of the existing evidence comes from ecological analyses, cross-sectional studies, or cohort studies with short follow-up periods, and the results are inconsistent (30–32).

Health disparities by SES are pronounced in Central and Eastern Europe, yet relatively few studies have focused on older populations in this region (33–36). Studying Eastern European populations is intriguing because they generally have shorter life expectancies and have experienced revolutionary political and economic reforms that have led to rapid changes in social structures (37, 38). A recent study utilizing harmonized data from 37 populations, including six from Central and Eastern Europe, indicated that the magnitude of social inequalities in PF was related to the economic strength of the country (39). In this report, based on a Polish cohort from the HAPIEE (Health, Alcohol and Psychosocial Factors in Eastern Europe) study, we hypothesized that upward social mobility is associated with a slower decline in PF compared to stable low SES and that downward social mobility is associated with a faster decline compared to consistently high SES. Specifically, we aimed to assess (1) the trajectories of PF between middle and old age stratified by sex, (2) the socioeconomic gradient in the change of PF, and (3) the relationship between social mobility and changes in PF.

## 2. Methods

### 2.1. Data and study design

The present analysis was conducted within the Polish arm of the HAPIEE study. This multicenter prospective cohort study aims to investigate the psychosocial and dietary factors that contribute to cardiovascular diseases (CVD) and other chronic conditions in Central and Eastern Europe. Detailed information regarding the study design and methods has been previously published (40). The present analysis involved a random sample of permanent residents of Krakow between the ages of 45 and 69, selected from the registry of residents. The exclusion criterion was a lack of informed consent. At the baseline, from 2002 to 2005, a total of 10,728 women and men underwent examination, with a participation rate of 62%. Adult SES and PF were assessed for all participants, and potential confounding factors and covariates were measured. Childhood SES and social mobility were also assessed at the baseline survey using a retrospectively focused questionnaire. To assess the trajectories of PF, participants from the baseline survey were followed up through face-to-face re-examinations and three postal surveys, spanning a period of up to 20 years (with an average follow-up of 18 years).

### 2.2. Physical functioning score

In the HAPIEE Study, the assessment of PF was conducted using a subset of the SF-36 scale, which consists of 10 questions related to activities involving vigorous and moderate physical exertion, lifting groceries, mobility, and self-care tasks (41). Participants were asked to rate themselves as “limited a lot,” “limited a little,” or “not limited at all” for each item. A composite score ranging from 0 to 100 was calculated, with a higher score indicating better PF. The PF score was assessed five times during the follow-up period. The first assessment took place at baseline between 2002 and 2005, the second assessment between 2006 and 2008, the third assessment between 2009 and 2010, the fourth assessment between 2012 and 2014, and the final assessment between 2020 and 2022. For inclusion in the present analysis, valid PF data had to be available for the first and last examinations, as well as for at least two out of the three intermediate assessments.

### 2.3. Socioeconomic status

The information regarding SES was collected at baseline (2002–2005) for both childhood SES (using a retrospective manner) and adulthood SES. Childhood SES was determined by considering the educational level of both parents (incomplete primary or no formal education, primary, vocational, secondary, or university) and information about housing standards at around the age of 10 (availability of cold tap water, hot tap water, radio, fridge, own kitchen, and own toilet). These factors were combined into an index to measure the amenities experienced during childhood.

Adulthood SES was assessed using five items: educational attainment, professional activity, household amenities, and current financial situation. The details of how the SES index was constructed have been previously described (42), and a summary is provided below.

A two-step clustering algorithm was employed to classify participants into homogeneous groups of low, middle, or high SES for both childhood and adulthood. The classification was based on reasonable evidence of the cluster structure, indicated by the silhouette coefficient s(i) of 0.51 (43). Based on the SES category in childhood and adulthood, four social mobility categories were generated for each participant: (1) always low SES, (2) downward mobility if the childhood SES was middle or high but adulthood SES was low, (3) upward mobility if the childhood SES was low but adulthood SES was middle or high, and (4) always middle or high SES. Supplementary Table 1 presents descriptive statistics for the SES clusters and the distribution of characteristics used to construct the SES index.

### 2.4. Covariates

During the baseline examination (2002–2005), a standardized questionnaire was administered to collect information on age, gender, education, marital status, and smoking habits. Body weight and height were measured to calculate the body mass index (BMI, kg/m^2^). Participants were asked about any self-reported diagnosis or hospitalization for spine or joint problems in the past year. Consumption of alcohol was assessed using the graduated frequency questionnaire (GFQ). By assessing drinking frequency and amounts consumed, the total annual alcohol consumption was calculated. It was assumed that 100 mL of beer, wine, and spirits contained 4, 10, or 36 g of ethanol, respectively (44).

### 2.5. Statistical analysis

Continuous variables were reported as means with standard deviations (SD). The normal distribution of variables was tested using the Shapiro–Wilk test. Categorical variables were presented as percentages. The Chi-square test was used to compare the distribution of categorical variables. The unpaired Student’s *t*-test was performed to assess differences between groups for numerical variables.

Given that the PF score was assessed at least four times during the follow-up, the Generalized Estimating Equations (GEE) technique was employed to model the longitudinal data (45). This approach allowed for the assessment of changes in the PF score over time and comparisons of the PF score trajectories across different SES categories. In the GEE models, the PF scores at each time point were the dependent variables. Considering the numerical nature of the PF score, the “Identity link” and unstructured-free estimation were used to account for within-subject correlation. Two sets of models were performed, separately for childhood SES, adulthood SES, and social mobility: Model A included age only, and Model B included additional adjustments for marital status, BMI, smoking status, spine/joint problems, and annual alcohol consumption.

The results of the GEE models were reported as coefficients with standard errors (SE) for differences at baseline and for differences in the trajectories of the PF score during the follow-up (interaction with time), presented as the decline per 1 year. Statistical analyses were performed using IBM Corp. software released in 2021 (IBM SPSS Statistics for Windows; version 28.0.; IBM Corp, Armonk, NY, United States) or R Core Team (2021). (R: A language and environment for statistical computing. R Foundation for Statistical Computing, Vienna, Austria). *p* < 0.05 was accepted as statistically significant (46).

## 3. Results

A total of 1,116 men and 1,178 women were included in the analysis, all of whom completed at least four examinations, including the first and last ones. Supplementary Figure 1 provides detailed information on the sample recruitment process.

The participants included in the analysis were younger [mean (SD) age of 56 (6.4) years vs. 58 (7.1) years], had higher levels of education (42% vs. 25% with university-level education), were more frequently married or cohabiting (80.6% vs. 75.0%), and had a lower prevalence of smoking (24.3% vs. 34.2%) compared to the remaining participants from the baseline examination (2002–2005) of the entire sample participating in the Polish part of the HAPIEE study. There was no difference in gender distribution (51.4% men vs. 51.2% women; Supplementary Table 2).

Within the analytical sample, men were slightly older than women and had higher rates of professional activity and were married or cohabiting. There was almost no difference in current smoking rates, but men had a higher frequency of past smoking. Notably, there was a significant difference in alcohol consumption between men and women (the median consumption was over eight times larger). Men also had slightly higher mean BMI and a higher prevalence of spine/joint problems (Table 1).

**Table 1:** Characteristics of the studied group.

	Men	Women		All
	*N* = 1,116	*N* = 1,178	*p*	
PF score (first examination), mean (SD)	89 (15.1)	83 (18)	<0.001	86 (16.94)
PF score (second examination), mean (SD)	90 (15)	85 (17)	<0.001	87 (16.28)
PF score (third examination), mean (SD)	82 (21.2)	73 (23.2)	<0.001	77 (22.68)
PF score (fourth examination), mean (SD)	79 (21.7)	70 (24.1)	<0.001	74 (23.4)
PF score (fifth examination), mean (SD)	71 (26.6)	64 (27.7)	<0.001	67 (27.33)
Age [years], mean (SD)	57 (6.5)	56 (6.2)	<0.001	56 (6.4)
Married or cohabiting, *n* (%)	1,024 (92.0%)	821 (70.0%)	<0.001	1,845 (80.6%)
**Childhood SES, *n* (%)**				
Low	388 (35.4%)	394 (33.9%)		782 (34.6%)
Middle	388 (35.4%)	409 (35.2%)	0.65	797 (35.3%)
High	321 (29.3%)	359 (30.9%)		680 (30.1%)
**Adulthood SES, *n* (%)**				
Low	186 (16.7%)	284 (24.1%)		470 (20.5%)
Middle	250 (22.4%)	261 (22.2%)	<0.001	511 (22.3%)
High	680 (60.9%)	633 (53.7%)		1,313 (57.2%)
**Social mobility, *n* (%)**				
Always low SES	67 (6.2%)	104 (9.1%)		171 (7.7%)
Downward mobility	109 (10.1%)	166 (14.5%)		275 (12.4%)
Upward mobility	314 (29.1%)	286 (25.0%)	0.002	600 (27.0%)
Always middle or high SES	591 (54.7%)	589 (51.4%)		1,180 (53.1%)
**Smoking status, *n* (%)**				
Current	266 (23.9%)	290 (24.7%)		556 (24.3%)
Former	452 (40.7%)	293 (24.9%)	<0.001	745 (32.6%)
Never	394 (35.4%)	593 (50.4%)		987 (43.1%)
BMI [kg/m^2^] mean (SD)	27.7 (3.73)	27.4 (4.56)	0.049	27.6 (4.18)
Spine/joint problems, *n* (%)	257 (23.1%)	228 (19.5%)	0.04	485 (21.2%)
Annual alcohol consumption [g], median (Q1–Q3)	840 (120–2,750)	100 (0–430)	<0.001	300 (0–1,530)

Regarding childhood SES, there were no differences in the distribution by SES category between men and women. Each of the three categories was equally represented by both sexes. However, in adulthood, a higher proportion of women had low SES (24.1% vs. 16.7%), while a higher proportion of men had high SES (60.9% vs. 53.7%). This difference reflects the fact that among women, the categories of always low and downward social mobility were more frequent compared to men. On the other hand, the proportions of participants in the categories of always middle or high and upward mobility were higher in men than in women.

At the baseline examination (2002–2005), the mean PF score was higher in men compared to women (89 vs. 83 points). Over time, there was a decline in PF scores in both sexes (Supplementary Figure 2). However, there was no significant difference in the trajectories of PF scores between men and women (*p* for interaction = 0.70). The decline in PF scores over time was consistent across all subsequent statistical models.

Table 2 presents the differences in baseline PF scores and the trajectories of PF scores according to childhood SES, adulthood SES, and social mobility categories. At the baseline (2002–2005), after adjusting for age, men with middle SES in childhood had a lower mean PF score compared to those with low SES in childhood by 3.2 points. On the other hand, women with high SES in childhood had a higher mean PF score compared to those with low SES by 4.2 points. SES in adulthood and social mobility were significantly and positively associated with PF scores in both sexes.

**Table 2:** Age-adjusted differences in physical functioning (PF) score at baseline and in trajectories of PF score by childhood SES, adulthood SES, and social mobility.

	Men		Women	
	Difference^a^ (SE)	*p*	Difference^a^ (SE)	*p*
**Baseline PF**				
Low childhood SES	Reference		Reference	
Middle childhood SES	**−3.2 (0.95)**	**0.002**	1.0 (1.04)	0.35
High childhood SES	1.1 (0.89)	0.33	**4.2 (0.97)**	**0.001**
**Difference in the decline of PF (per year)**				
Low childhood SES	Reference		Reference	
Middle childhood SES	**0.24 (0.112)**	**0.02**	−0.02 (0.126)	0.86
High childhood SES	**0.26 (0.122)**	**0.023**	**0.28 (0.119)**	**0.016**
**Baseline PF**				
Low adulthood SES	Reference		Reference	
Middle adulthood SES	**7.3 (1.19)**	**<0.001**	**10.5 (1.39)**	**<0.001**
High adulthood SES	**9.2 (1.26)**	**<0.001**	**11.2 (1.1)**	**<0.001**
**Difference in the decline of PF (per year)**				
Low adulthood SES	Reference		Reference	
Middle adulthood SES	**0.30 (0.178)**	**0.03**	**−0.29 (0.134)**	**0.03**
High adulthood SES	**0.32 (0.148)**	**0.008**	**0.51 (0.128)**	**<0.01**
**Baseline SES**				
Always low SES	Reference		Reference	
Downward mobility	−3.3 (2.53)	0.13	1.9 (2.11)	0.32
Upward mobility	**7.2 (2.07)**	**0.002**	**10.5 (1.86)**	**<0.001**
Always middle or high SES	**6.2 (2.02)**	**0.007**	**12.8 (1.77)**	**<0.001**
**Difference in the decline of PF (per year)**				
Always low SES	Reference		Reference	
Downward mobility	0.43 (1.879)	0.06	−0.04 (0.200)	0.82
Upward mobility	0.29 (0.198)	0.185	0.14 (0.178)	0.42
Always middle or high SES	**0.47 (0.199)**	**0.014**	**0.31 (0.160)**	**0.048**

^a^Age-adjusted coefficient of the GEE model; SE, standard error; significant results are bolded.

In women, compared to those with low SES in adulthood, those with middle and high SES in adulthood had higher mean PF scores by more than 10.5 and 11.2 points, respectively. Similarly, compared to those with always low SES, women with upward social mobility and women who always had middle or high SES had higher PF scores by approximately 10.5 and 12.8 points, respectively.

In men, the differences in PF scores according to adulthood SES and social mobility categories were similar to those observed in women although the magnitude of the differences was slightly smaller (7.3 and 9.2 points in the case of adulthood SES and 7.2 and 6.2 points in the case of social mobility categories).

The trajectories of PF between the baseline and final examinations varied significantly according to childhood SES, adulthood SES, and social mobility categories in both men and women.

In men, those with middle and high SES in childhood experienced a slower decline in PF compared to those with low childhood SES. Similarly, men with middle and high SES in adulthood experienced a slower decline in PF compared to those with low adulthood SES. Regarding social mobility, only men who had always high or middle SES demonstrated a slower decline in PF compared to those who had always low SES (by 0.47 points per year).

Among women, those with high SES in childhood (but not those with middle childhood SES) had a slower decline in PF compared to those with low childhood SES. Women with middle adulthood SES and women with high adulthood SES also experienced a slower decline in PF compared to those with low adulthood SES. Similar to men, in the social mobility analyses, only women with always high or middle SES demonstrated a slower decline in PF compared to those who had always low SES (by 0.31 points per year).

**Figure 1:**
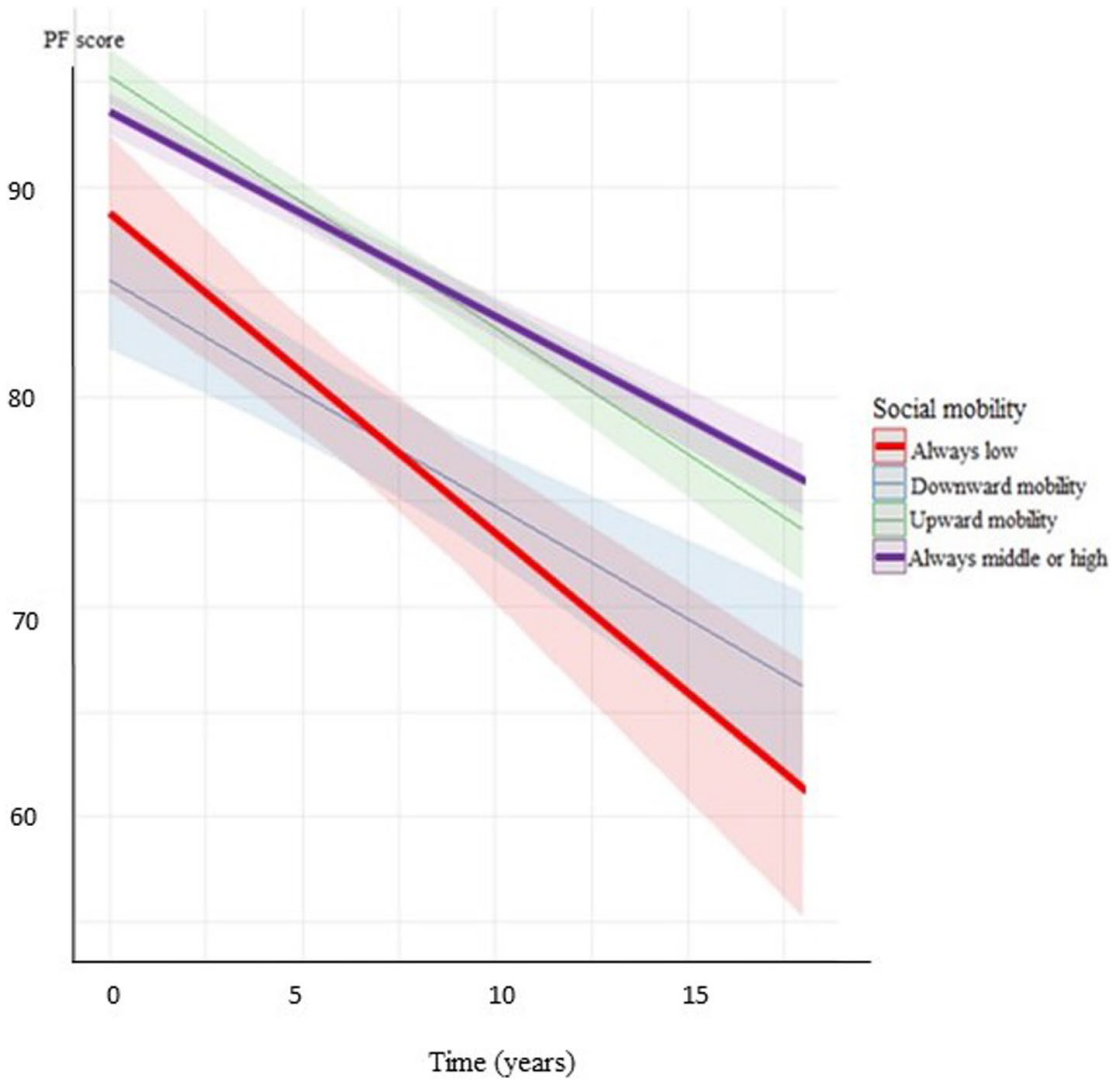
Decline of PF score by the categories of social mobility in men (adjusted for age, marital status, BMI, smoking status, spine/joint problems, and annual alcohol consumption).

**Figure 2:**
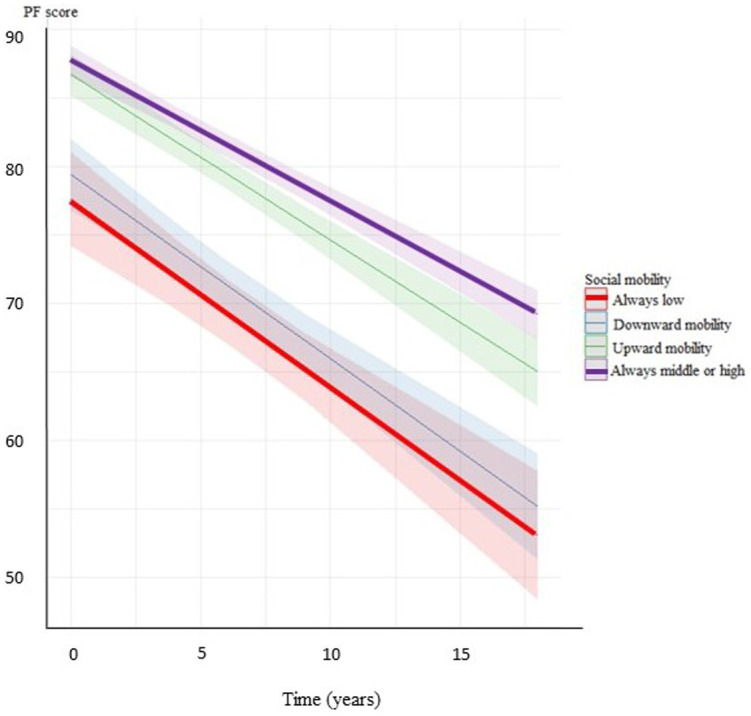
Decline of PF score by the categories of social mobility in women (adjusted for age, marital status, BMI, smoking status, spine/joint problems, and annual alcohol consumption).

Additional adjustments for covariates such as marital status, history of spine/joint problems, BMI, smoking, and alcohol consumption did not significantly change most of the results. The only exception was the difference in the trajectories of PF between men with middle adulthood SES and men with low adulthood SES, which lost significance after adjustment for covariates although the change in the estimate was minimal (Table 3; Figures 1, 2).

**Table 3:** Differences in physical functioning (PF) score at baseline and in trajectories of PF score by childhood SES, adulthood SES, and social mobility (adjusted for all covariates).

	Men		Women	
	Difference^b^ (SE)	*p*	Difference^b^ (SE)	*p*
**Baseline PF**				
Low childhood SES	Reference		Reference	
Middle childhood SES	**−3.5 (1.03)**	**0.006**	0.7 (1.09)	0.51
High childhood SES	0.1 (1.10)	0.94	**2.3 (1.14)**	**0.04**
**Difference in the decline of PF (per year)**				
Low childhood SES	Reference		Reference	
Middle childhood SES	**0.28 (0.106)**	**0.009**	−0.01 (0.113)	0.91
High childhood SES	**0.32 (0.123)**	**0.004**	**0.33 (0.116)**	**0.005**
**Baseline PF**				
Low adulthood SES	Reference		Reference	
Middle adulthood SES	**6.9 (1.37)**	**<0.001**	**9.3 (1.32)**	**<0.001**
High adulthood SES	**7.7 (1.17)**	**<0.001**	**8.5 (1.11)**	**<0.001**
**Difference in the decline of PF (per year)**				
Low adulthood SES	Reference		Reference	
Middle adulthood SES	0.25 (0.138)	0.072	**−0.30 (0.134)**	**0.026**
High adulthood SES	**0.39 (0.120)**	**0.001**	**0.50 (0.112)**	**<0.001**
**Baseline SES**				
Always low SES	Reference		Reference	
Downward mobility	−3.1 (2.20)	0.16	1.8 (1.89)	0.344
Upward mobility	**6.6 (1.90)**	**<0.001**	**9.1 (1.73)**	**<0.001**
Always middle or high SES	**4.8(1.83)**	**0.009**	**10.1 (1.62)**	**<0.001**
**Difference in the decline of PF (per year)**				
Always low SES	Reference		Reference	
Downward mobility	0.44 (0.226)	0.06	0.02 (0.196)	0.94
Upward mobility	0.32 (0.196)	0.100	0.16 (0.179)	0.38
Always middle or high SES	**0.55 (0.188)**	**0.003**	**0.34 (0.166)**	**0.04**

^b^Coefficient of the GEE model, adjusted for age, marital status, BMI, smoking status, spine/joint problems, and annual alcohol consumption; SE, standard error; significant results are bolded.

## 4. Discussion

In this population-based cohort study conducted in Central and Eastern Europe, we observed significant differences in PF based on SES indicators measured at baseline. Moreover, we identified variations in the decline of PF over time among different SES categories. The analysis of trajectories suggests that while health disparities related to childhood SES may diminish during middle age if people improve their socioeconomic position, these disparities may reappear as a delayed effect during the transition from middle to old age.

Previous studies have proposed that upward social mobility, along with the stress associated with climbing the social ladder, could have harmful effects on health (47–49). However, there is also evidence that upward social mobility was associated with slower biological aging and people with more socioeconomic resources appeared biologically younger than people of the same chronological age with fewer socioeconomic resources (50). A study conducted in the United States examined healthy aging using blood chemistry and DNA methylation measures of biological aging in a national sample of older adults. The study found that individuals who grew up in socioeconomically disadvantaged families and those who accumulated less wealth over their lives experienced faster biological aging compared to those who grew up in socioeconomically advantaged families. Additionally, participants who experienced upward social mobility demonstrated slower biological aging in later life compared to non-mobile or downwardly mobile participants (51). Similarly, a British study revealed that DNA methylation age acceleration was associated with both early-life SES and adult SES. Furthermore, upward mobility was linked to lower accelerated aging compared to individuals who experienced disadvantages throughout their life course (52).

The findings from our previous study on disparities in PF in three populations from Central and Eastern Europe provided support for the cumulative advantage/disadvantage hypothesis. High SES was associated with better PF at baseline and a slower decline during the follow-up period (36). Our results contradict some previous studies that suggested health costs associated with upward social mobility (53–55). They are also inconsistent with the findings of a recent analysis of SHARE data of people aged 50 years and older, which demonstrated a reciprocal relationship between SES and health. In that study, the influence of health selection (reverse causation) was stronger than the causal mechanisms through which SES could affect PF and other objectively measured health indices (social causation). Additionally, higher baseline SES, which was associated with less SES increase and better initial health, was related to less improvement in health (17, 56). However, our findings align with a joint analysis of longitudinal SHARE and ESLA data, which showed that participants’ health significantly depended on their prior status, and social causation played a prominent role in the transition from adulthood to old age (57).

Overall, our study confirmed earlier findings that psychological and social factors play an important role in slowing down the age-related decline in PF and that lower social status is associated with accelerated aging (26, 58). In our study, this difference was reflected in the trajectory of PF between individuals with always middle or high SES and those with always low SES. Our results also align with an American cross-sectional study, which demonstrated that stable low SES was associated with worse PF assessed using objective measures such as aerobic endurance, gait speed, and lower body strength. Moreover, the study found no significant difference in PF between upward mobility and stable high or middle SES groups, while stable low SES and downward social mobility were strongly and equally associated with poorer PF compared to upward mobility (31).

In a previous Polish study involving over 2,500 adults across a wide age range, participants from low social classes reported worse self-rated health. However, upward social mobility was only related to better psychological well-being, not self-rated health. The differences between that study and our findings, where upward social mobility was significantly associated with better PF, could be explained by variations in study design (cross-sectional vs. longitudinal), outcomes assessed (self-rated health vs. PF), and the method used to classify SES (based on occupational status only in the previous study) (35). Additionally, the strength of the relationship between SES and self-rated health is known to depend on the SES indicator used (59), and the results of a Swedish study suggest a strong mediation effect of working conditions on the association between social class and physical impairment in older age (60).

### 4.1. Limitations

There are several limitations to our study that should be taken into consideration. Firstly, the measures of childhood and adulthood SES were self-reported, which introduces the possibility of reporting bias and misclassification. Additionally, the selected measures may not capture the full range of all relevant SES indicators that could potentially influence health and PF.

Secondly, we lacked prospective data on SES and PF over the entire life course. While childhood circumstances and educational attainment tend to remain stable since early adulthood, material conditions may have changed during the follow-up period. Moreover, the assessment of covariates at a single time point is a concern, as these factors may have fluctuated over the course of nearly two decades. In addition, the use of interviews to collect these data may introduce biases. For example, the assessment of alcohol intake relied on a questionnaire method, which is known to underestimate actual consumption. However, it should be noted that this method is reliable in ranking participants based on their alcohol consumption (61).

Thirdly, there may be concerns regarding the assessment of PF. Although we used a standardized method validated for the Polish population throughout the follow-up, the interview-based assessment could still be influenced by local culture, social roles, cognitive abilities, and other factors.

Finally, the analyses included a healthier subset of the population from which the sample was selected. This is due to both natural attrition, as over 30% of participants did not survive or were lost to follow-up until the final examination, and the low participation rate in at least four assessments of PF. In a previous report, we found that non-participants were at a higher risk of death (62). Therefore, generalizing the findings to the entire general population may be uncertain.

### 4.2. Strengths

This study examined a general population sample from Central Europe, a region with relatively lower life expectancy compared to Western Europe and the United States (37). This region has received less attention in terms of studying the relationships between health and psycho-socioeconomic factors. The measurement of PF was conducted using a well-established method that has been validated for the Polish population (41). The repeated measurements of PF using the same method allowed us to assess longitudinal trajectories of PF over an average period of 18 years, spanning from middle to old age. Furthermore, we were able to account for important potential confounding factors in our analyses, including alcohol consumption, which we have previously observed to be a significant determinant of PF trajectories (63, 64).

## 5. Conclusion

As the proportion of older people in the population continues to rise, the issue of declining PF with age has become a critical public health concern. This cohort study revealed that lower SES and downward social mobility were associated with worse PF, while upward social mobility appeared to counteract the negative effects of low childhood SES. Furthermore, in addition to the cross-sectional associations observed at baseline, higher SES was significantly linked to a slower decline in PF over an 18-year follow-up period. Addressing childhood social inequalities and maintaining high SES levels appear to be crucial in mitigating the decline in PF during old age and could help to maintain the self-sufficiency and social engagement of older people. Future long-term observational studies should focus on investigating the extent to which reducing childhood inequalities could impact the decline of PF in adulthood and old age.

## Data availability statement

The raw data supporting the conclusions of this article will be made available by the authors, without undue reservation.

## Author contributions

AP, MP, MK, AD, and MB contributed to the intellectual conceptualization of this study. AP an MP designed the paper and wrote the first draft and finalized the paper. MP performed the statistical analyses. AP and MB jointly designed the HAPIEE study. AD and MK participated in the coordination of data collection. All authors contributed to the article and approved the submitted version.

## Funding

This work was supported by a grant from the Polish National Science Centre (grant 2018/29/B/NZ7/02118). The HAPIEE study was funded by the Welcome Trust (grant WT064947 and WT081081), the US National Institute of Aging (grant R01 AG23522), and the MacArthur Foundation. MB’s contribution was supported by the European Union’s Horizon 2020 Research and Innovation Program projects CETOCOEN Excellence (grant agreement 857560) and R-Exposome Chair (grant agreement 857487).

## Conflict of interest

The authors declare that the research was conducted in the absence of any commercial or financial relationships that could be construed as a potential conflict of interest.

## Publisher’s note

All claims expressed in this article are solely those of the authors and do not necessarily represent those of their affiliated organizations, or those of the publisher, the editors and the reviewers. Any product that may be evaluated in this article, or claim that may be made by its manufacturer, is not guaranteed or endorsed by the publisher.

## References

[1] Cosco TD Prina AM Perales J Blosson CMS Brayne C. Operational definitions of successful aging: a systematic review. Int Psychogeriatr. (2014) 26:373–81. doi: 10.1017/S1041610213002287, PMID: 24308764

[2] Kozela M Pająk A Szafraniec K Ayuso-Mateos JL Bobak M Lu W. ATHLOS healthy aging scale score as the predictor of all-cause mortality in Poland and Czechia. Front Public Health. (2023) 11:1114497. doi: 10.3389/fpubh.2023.1114497, PMID: 37006584 PMC10061126

[3] Fried LP Ferrucci L Darer J Williamson JD Anderson G. Untangling the concepts of disability, frailty, and comorbidity: implications for improved targeting and care. J Gerontol Series A. (2004) 59:255–63. doi: 10.1093/gerona/59.3.M2552, PMID: 15031310

[4] Feinstein JS. The relationship between socioeconomic status and health: a review of the literature. Milbank Q. (1993) 71:279–322. doi: 10.2307/3350401, PMID: 8510603

[5] Marmot M Ryff CD Bumpass LL Marks NF. Social inequalities in health: next questions and converging evidence. Soc Sci Med. (1997) 44:901–10. doi: 10.1016/s0277-9536(96)00194-3, PMID: 9080570

[6] Bobak M Pikhart H Rose R Marmot M. Socioeconomic factors, material inequalities, and perceived control in self-rated health: cross-sectional data from seven post-communist countries. Soc Sci Med. (2000) 51:1343–50. doi: 10.1016/s0277-9536(00)00096-4, PMID: 11037221

[7] Ross CE Wu CL. Education, age, and the cumulative advantage in health. J Health Soc Behav. (1996) 37:104–20. doi: 10.2307/2137234, PMID: 8820314

[8] Kim J Durden E. Socioeconomic status and age trajectories of health. Soc Sci Med. (2007) 65:2489–502. doi: 10.1016/j.socscimed.2007.07.022 17765375

[9] Chandola T Ferrie J Sacker A Marmot M. Social inequalities in self reported health in early old age: follow-up of prospective cohort study. BMJ. (2007) 334:990–3B. doi: 10.1136/bmj.39167.439792.55, PMID: 17468119 PMC1867907

[10] Kim J Richardson V. The impact of socioeconomic inequalities and lack of health insurance on physical functioning among middle-aged and older adults in the United States. Health Soc Care Community. (2012) 20:42–51. doi: 10.1111/j.1365-2524.2011.01012, PMID: 21733029

[11] Zaninotto P Sacker A Head J. Relationship between wealth and age trajectories of walking speed among older adults: evidence from the English longitudinal study of ageing. J Gerontol A Biol Sci Med Sci. (2013) 68:1525–31. doi: 10.1093/gerona/glt058, PMID: 23682157 PMC3814237

[12] Taylor MG. Capturing transitions and trajectories: the role of socioeconomic status in later life disability. J Gerontol B Psychol Sci Soc Sci. (2010) 65:733–43. doi: 10.1093/geronb/gbq018, PMID: 20427460 PMC2954323

[13] Gerstorf D Ram N Lindenberger U Smith J. Age and time-to-death trajectories of change in indicators of cognitive, sensory, physical, health, social, and self-related functions. Dev Psychol. (2013) 49:1805–21. doi: 10.1037/a0031340, PMID: 23356526

[14] Koster A Bosma H Broese van Groenou MI Kempen GIJM Penninx BWJH van Eijk JTM. Explanations of socioeconomic differences in changes in physical function in older adults: results from the longitudinal aging study Amsterdam. BMC Public Health. (2006) 6:244. doi: 10.1186/1471-2458-6-244, PMID: 17022819 PMC1621070

[15] House JS Lepkowski JM Kinney AM Mero RP Kessler RC Herzog AR. The social stratification of aging and health. J Health Soc Behav. (1994) 35:213–34. doi: 10.2307/2137277 7983335

[16] Lynch SM. Cohort and life-course patterns in the relationship between education and health: a hierarchical approach. Demography. (2003) 40:309–31. doi: 10.1353/dem.2003.0016, PMID: 12846134

[17] Ahrenfeldt LJ Möller S. The reciprocal relationship between socioeconomic status and health and the influence of sex: a European SHARE-analysis based on structural equation modeling. Int J Environ Res Public Health. (2021) 18:5045. doi: 10.3390/ijerph18095045, PMID: 34068750 PMC8126237

[18] Galobardes B Shaw M Lawlor DA Lynch JW Davey SG. Indicators of socioeconomic position (part 1). J Epidemiol Community Health. (2006) 60:7–12. doi: 10.1136/jech.2004.023531, PMID: 16361448 PMC2465546

[19] Belsky DW Caspi A Cohen HJ Kraus WE Ramrakha S Poulton R. Impactofearlypersonal-history characteristics on the pace of aging: implications for clinical trials of therapies to slow aging and extend health span. Aging Cell. (2017) 16:644–51. doi: 10.1111/acel.12591, PMID: 28401731 PMC5506399

[20] Marini S Davis KA Soare TW Zhu Y Suderman MJ Simpkin AJ. Adversity exposure during sensitive periods predicts accelerated epigenetic aging in children. Psychoneuroendocrinology. (2020) 113:104484. doi: 10.1016/j.psyneuen.2019.104484, PMID: 31918390 PMC7832214

[21] Moor I Spallek J Richter M. Explaining socioeconomic inequalities in selfrated health: a systematic review of the relative contribution of material, psychosocial and behavioural factors. J Epidemiol Community Health. (2017) 71:565–75. doi: 10.1136/jech-2016-207589, PMID: 27682963

[22] Herrmann J Vogel M Pietzner D Kroll E Wagner O Schwarz S. Factors associated with the emotional health of children: high family income as a protective factor. Eur Child Adolesc Psychiatry. (2018) 27:319–28. doi: 10.1007/s00787-017-1049-0, PMID: 28942492

[23] Poulaina T Vogela M Kiess W. Review on the role of socioeconomic status in child health and development. Curr Opin Pediatr. (2020) 32:308–14. doi: 10.1097/MOP.0000000000000876 31895161

[24] Dathan-Stumpf A Vogel M Rieger K Thiery J Kiess HA. Serum lipid levels were related to socio-demographic characteristics in a German population-based child cohort. Acta Paediatr. (2016) 105:e360–7. doi: 10.1111/apa.13438, PMID: 27096544

[25] Rieger K Vogel M Engel C Ceglarek U Harms K Wurst U. Does physiological distribution of blood parameters in children depend on socioeconomic status? Results of a German cross-sectional study. BMJ Open. (2018) 8:019143:e019143. doi: 10.1136/bmjopen-2017-019143, PMID: 29500207 PMC5855248

[26] Steptoe A Zaninotto P. Lower socioeconomic status and the acceleration of aging: an outcome-wide analysis. Proc Natl Acad Sci U S A. (2020) 117:14911–7. doi: 10.1073/pnas.1915741117, PMID: 32541023 PMC7334539

[27] Laurie M Corna A. Life course perspective on socioeconomic inequalities in health: a critical review of conceptual frameworks. Adv Life Course Res. (2013) 18:150–9. doi: 10.1016/j.alcr.2013.01.002 24796266

[28] Cutler DM Lleras-Muney A Vogl T. Socioeconomic status and health: Dimensions and mechanisms. (2008). In: *NBER Working Paper No. 14333, 2008*.

[29] Sorokin PA. Social and cultural mobility. New York: Free Press (1959).

[30] Schmengler H Margot PM Stevens GWJM Kunst AE Delaruelle K Dierckenes M. Socioeconomic inequalities in adolescent health behaviours across 32 different countries—the role of country-level social mobility. Soc Sci Med. (2022) 310:115289. doi: 10.1016/j.socscimed.2022.115289, PMID: 35994878

[31] Noppert GA Brown CS Chanti-Ketter M Hall KS Newby LK Cohen HJ. The impact of multiple dimensions of socioeconomic status on physical functioning across the life course. Gerontol Geriatr Med. (2018) 4:233372141879402–8. doi: 10.1177/2333721418794021, PMID: 30186891 PMC6113730

[32] Torres JM Rizzo S Wong R. Lifetime socioeconomic status and late-life health trajectories: longitudinal results from the Mexican health and aging study. J Gerontol B Psychol Sci Soc Sci. (2018) 73:gbw048–360. doi: 10.1093/geronb/gbw048, PMID: 27140821 PMC5927147

[33] Kozela M Polak M Stepaniak U Bobak M Pająk A. Changes in socioeconomic status as predictors of cardiovascular disease incidence and mortality: a 10-year follow-up of a polish-population-based HAPIEE cohort. Int J Environ Res Public Health. (2022) 19:15411. doi: 10.3390/ijerph192215411, PMID: 36430130 PMC9693797

[34] Słomczynski KM Wysmułek I. Social inequality and the life course: Poland’s transformative years, 1988–2013. Warsaw, Poland: IFiS Publishers (2016).

[35] Zelinska O Gugushvili A Bulczak G. Social mobility, health and wellbeing in Poland. Front Sociol. (2021) 6:736249. doi: 10.3389/fsoc.2021.736249, PMID: 34901260 PMC8656426

[36] Hu Y Pikhart H Pająk A Kubínová R Malyutina S Besala A. Education, material condition and physical functioning trajectories in middle-aged and older adults in central and Eastern Europe: a cross-country comparison. J Epidemiol Commun Health. (2016) 70:1128–35. doi: 10.1136/jech-2015-206548, PMID: 27194710 PMC5541176

[37] World Health Statistics 2022: Monitoring health for the SDGs, sustainable development goals Geneva (2022). Available at: https://www.who.int/data/gho/publications/world-health-statistics (Accessed May 2023).

[38] Healey NM. The transition economic of central and eastern Europe: a political, economic, social and technological analysis. Columbia J World Bus. (1994) 29:62–70. doi: 10.1016/0022-5428(94)90020-5

[39] Stefler D Prina M Wu YT Sánchez-Niubò A Lu W Haro JM. Socioeconomic inequalities in physical and cognitive functioning: cross-sectional evidence from 37 cohorts across 28 countries in the ATHLOS project. J Epidemiol Community Health. (2021) 75:980–6. doi: 10.1136/jech-2020-214714, PMID: 33649052

[40] Peasey A Bobak M Kubinova R Malyutina S Pajak A Tamosiunas A. Determinants of cardiovascular disease and other non-communicable diseases in central and Eastern Europe: rationale and design of the HAPIEE study. BMC Public Health. (2006) 6:255. doi: 10.1186/1471-2458-6-255, PMID: 17049075 PMC1626086

[41] Ware JE. SF-36 health survey update. Spine. (2000) 25:3130–9. doi: 10.1097/00007632-200012150-00008 11124729

[42] Polak M Szafraniec K Kozela M Wolfshaut-Wolak R Bobak M Pająk A. Socioeconomic status and pulmonary function, transition from childhood to adulthood: cross-sectional results from the polish part of the HAPIEE study. BMJ Open. (2019) 9:e022638. doi: 10.1136/bmjopen-2018-022638, PMID: 30782683 PMC6340009

[43] Bacher J Wenzig K Vogler M. (2004) SPSS TwoStep cluster—a first evaluation. 2nd ed. Arbeitsund Diskussionspapiere/Universität Erlangen-Nürnberg, Sozialwissenschaftliches Institut, Lehrstuhl für Soziologie, 2004-2. Nürnberg: Universität Erlangen-Nürnberg, Wirtschafts- und Sozialwissenschaftliche Fakultät, Sozialwissenschaftliches Institut Lehrstuhl für Soziologie. Available at: https://nbn-resolving.org/urn:nbn:de:0168-ssoar-327153.

[44] Rehm J. Measururing quantity, frequency, and volume of drinking. Alcohol Clin Exp Res. (1998) 22:4S–14S. doi: 10.1097/00000374-199802001-00002 9603301

[45] Ballinger GA. Using generalized estimating equations for longitudinal data analysis. Organ Res Methods. (2004) 7:127–50. doi: 10.1177/1094428104263672

[46] R Core Team. R: A language and environment for statistical computing. Vienna, Austria: R Foundation for Statistical Computing (2021). Availabe at: https://www.R-project.org/.

[47] Colen CG Geronimus AT Bound J James SA. Maternal upward socioeconomic mobility and black–white disparities in infant birthweight. Am J Public Health. (2006) 96:2032–9. doi: 10.2105/AJPH.2005.076547, PMID: 17018818 PMC1751798

[48] Kuh D Ben-Shlomo Y Lynch J Hallqvist J Power C. Life course epidemiology. J Epidemiol Community Health. (2003) 57:778–83. doi: 10.1136/jech.57.10.778, PMID: 14573579 PMC1732305

[49] McEwen BS Gianaros PJ. Central role of the brain in stress and adaptation: links to socioeconomic status, health, and disease. Ann N Y Acad Sci. (2010) 1186:190–222. doi: 10.1111/j.1749-6632.2009.05331.x, PMID: 20201874 PMC2864527

[50] Raffington L Belsky DW Kothari M Malanchini M Tucker-Drob EM Harden KP. Socioeconomic disadvantage and the pace of biological aging in children. Pediatrics. (2021) 147:e2020024406. doi: 10.1542/peds.2020-024406, PMID: 34001641 PMC8785753

[51] Graf GH Zhang Y Domingue BW Harris KM Kothari M Kwon D. Social mobility and biological aging among older adults in the United States. PNAS Nexus. (2022) 1:1–10. doi: 10.1093/pnasnexus/pgac029, PMID: 35615471 PMC9123172

[52] Bao Y Gorrie-Stone T Hannon E Hughes A Andrayas A Neilson G. Social mobility across the lifecourse and DNA methylation age acceleration in adults in the UK. Sci Rep. (2022) 12:22284. doi: 10.1038/s41598-022-26433-2, PMID: 36566336 PMC9790005

[53] Chen E Miller GE Brody GH Lei M. Neighborhood poverty, college attendance, and diverging profiles of substance use and allostatic load in rural African American youth. Clin Psychol Sci. (2015) 3:675–85. doi: 10.1177/2167702614546639, PMID: 26366329 PMC4565724

[54] Brody GH Yu T Chen E Miller GE. Persistence of skin-deep resilience in African American adults. Health Psychol. (2020) 39:921–6. doi: 10.1037/hea0000945, PMID: 32597677 PMC8169037

[55] Gaydosh L Schorpp KM Chen E Miller GE Harris KM. College completion predicts lower depression but higher metabolic syndrome among disadvantaged minorities in young adulthood. Proc Natl Acad Sci U S A. (2018) 115:109–14. doi: 10.1073/pnas.1714616114, PMID: 29255040 PMC5776811

[56] Schwartz E Litwin H. The reciprocal relationship between social connectedness and mental health among older European adults: a SHARE-based analysis. J Gerontol B Psychol Sci Soc Sci. (2019) 74:694–702. doi: 10.1093/geronb/gbx131, PMID: 29126316 PMC6460342

[57] Hoffmann R Kröger H Pakpahan E. The reciprocal relationship between material factors and health in the life course: evidence from SHARE and ELSA. Eur J Ageing. (2018) 15:379–91. doi: 10.1007/s10433-018-0458-3, PMID: 30532675 PMC6250643

[58] Saadeh M Welmer AK Dekhtyar S Fratiglioni L Calderón-Larrañaga A. The role of psychological and social well-being on physical function trajectories in older adults. J Gerontol A Biol Sci Med Sci. (2020) 75:1579–85. doi: 10.1093/gerona/glaa114, PMID: 32384140 PMC7357580

[59] Moor I Kuipers MAG Lorant V Pförtner TK Kinnunen JM Rathmann K. Inequalities in adolescent self-rated health and smoking in Europe: comparing different indicators of socioeconomic status. J Epidemiol Community Health. (2019) 73:963–70. doi: 10.1136/jech-2018-211794, PMID: 31302594

[60] Pandey N Darin-Mattsson A Nilsen C. Working conditions mediate the association between social class and physical function in older age in Sweden: a prospective cohort study. BMC Public Health. (2020) 20:1360–10. doi: 10.1186/s12889-020-09431-9, PMID: 32887580 PMC7487473

[61] Gmel G Rehm J. Measuring alcohol consumption. Contemp Drug Probs. (2004) 31:467–540. doi: 10.1177/009145090403100304

[62] Topór-Madry R Bobak M Pajak A. 5-year mortality in respondents and nonrespondent for the cohort study of 20 000 randomly selected middle aged men and women the HAPIEE project. Eur J Prev Cardiol. (2012) 19:S71.

[63] Hu Y Pikhart H Kubinova R Malyutina S Pajak A Besala A. Alcohol consumption and longitudinal trajectories of physical functioning in central and Eastern Europe: a 10-year follow-up of HAPIEE study. J Gerontol A Biol Sci Med Sci. (2016) 71:1063–8. doi: 10.1093/gerona/glv233, PMID: 26748094 PMC4945885

[64] Hu Y Pikhart H Malyutina S Pajak A Kubinova R Nikitin Y. Alcohol consumption and physical functioning among middle-aged and older adults in central and Eastern Europe: results from the HAPIEE study. Age Ageing. (2015) 44:84–9. doi: 10.1093/ageing/afu083, PMID: 24982097 PMC4255613

