# Supplementary material for: Trajectories in physical functioning at older age in relation to childhood and adulthood SES and social mobility: a population-based cohort study
Source: Front Public Health. 2023 Sep 7;11:1228920. doi: 10.3389/fpubh.2023.1228920 (PMC10513394; doi:10.3389/fpubh.2023.1228920)
Supplement: Supplementary file 1 [file Data_Sheet_1.docx]

Supplementary Material

Article Title

Trajectories in physical functioning at older ages in relation to childhood and adulthood SES, and to social mobility: a population-based cohort study

**Andrzej Pająk^1,^ ^†^, Maciej Polak^1,^ ^†, *^, Magdalena Kozela^1^, Agnieszka Doryńska^2^, Martin Bobak^3, 4^**

^1^Department of Epidemiology and Population Studies, Institute of Public Health, Faculty of Health Sciences, Jagiellonian University Medical College.

^2^Department of Epidemiology, Cardiovascular Disease Prevention and Health Promotion, National Institute of Cardiology, Warsaw, Poland

^3^Research Centre for Toxic Compounds in the Environment (RECETOX), Masaryk University, Brno, Czech Republic

^4^Research Department of Epidemiology and Public Health, University College London, London, UK

† These authors contributed equally to this work and share first authorship

Corresponding Author
Maciej Polak

e-mail: maciej.1.polak@uj.edu.pl

**Supplementary Table 1**. SES clusters and distribution of characteristics used for SES index.

|  | **Childhood SES** | | | |
| --- | --- | --- | --- | --- |
| Variable |  | Low | Middle | High |
| The highest completed level of education of parents | Father |  |  |  |
|  | lower than secondary school | 771 (100%) | 592(75.5%) | 68 (10.1%) |
|  | secondary school | 0 | 161 (20.5%) | 306 (45.6%) |
|  | university degree | 0 | 31(4.0%) | 297 (44.3%) |
|  | Mather |  |  |  |
|  | lower than secondary school | 771 (100%) | 761 (97.4%) | 0 |
|  | secondary school | 0 | 22 (2.8%) | 542 (80.8%) |
|  | university degree | 0 | 1 (0.1%) | 129 (19.2%) |
| Index measuring amenities in childhood, calculated based on questions about which of the following items participants had at age 10 (cold tap water, hot tap water, radio, fridge, own kitchen and own toilet). Median (Q1-Q), range:0-6 | | 3 (2-4) | 4 (2-5) | 5 (4-6) |
|  | **Adulthood SES** | | | |
|  |  | Low | Middle | High |
| Highest completed level of education | lower than secondary school | 172 (36.6%) | 118 (23.1%) | 142 (10.8%) |
|  | secondary school | 226 (48.1%) | 217 (42.5%) | 454 (34.6%) |
|  | university degree | 72 (15.3%) | 176 (34.4%) | 717 (54.6%) |
| Current professional position | owner of a company | 0 | 0 | 173 (13.2%) |
|  | employed, | 82 (17.5%) | 0 | 1086 (82.7%) |
|  | retired or unemployed | 388 (82.6+%) | 511 (100%) | 54 (4.1%) |
| Index of household amenities based on the number of valuable items which a participant had in his household (microwave, washing machine, cottage house etc.). Median (Q1-Q3), range 0-12. | | 6 (4-7) | 7 (5-8) | 8 (6-9) |
| Current financial situation (based on two questions: | |  |  |  |
| “How often does it happen not to have enough money for food which you and your family need?” | all the time | 23 (4.9%) | 0 | 3 (0.2%) |
|  | often | 120 (25.5%) | 0 | 514 (1.07%) |
|  | sometimes | 165 (35.1%) | 0 | 76 (5.8%) |
|  | rarely | 109 (23.2%) | 0 | 124 (9.4%) |
|  | never | 53 (11.3%) | 511 (100%) | 1096 (83.5%) |
| Do you have any difficulties with paying bills | all the time | 36 (7.7%) | 0 | 11 (0.8%) |
|  | often | 90 (19.2%) | 0 | 36 (2.7%) |
|  | sometimes | 155 (33.0%) | 0 | 129 (9.8%) |
|  | rarely | 93 (19.8%) | 0 | 138 (10.5%) |
|  | never | 96 (20.4%) | 511 (100%) | 999 (76.1%) |

**Supplementary Table 2**. Distribution of age, sex education, smoking status and marital status by participation in the study

|  | Baseline examination n=10 728 | Included into the analysis  n=2924 | p |
| --- | --- | --- | --- |
| Age [years], mean (SD) | 58 (7.1) | 56 (6.4) | <0.001 |
| Women, n (%) | 4320 (51.2%) | 1178 (51.4%) | 0.9 |
| Education, n (%) |  |  | |
| Primary | 3073 (36.5%) | 432 (18.8%) |  |
| Secondary | 3255 (38.6%) | 897 (39.1%) | <0.001 |
| University | 2095 (24.9%) | 965 (42.1%) |  |
| Smoking status, n (%) |  |  | |
| Current | 2877 (34.2%) | 556 (24.3%) |  |
| Former | 2281 (27.1%) | 745 (32.6%) | <0.001 |
| Never | 3251 (38.7%) | 987 (43.1%) |  |
| Married or cohabiting, n (%) | 6314 (75.0%) | 1845 (80.6%) | <0.001 |


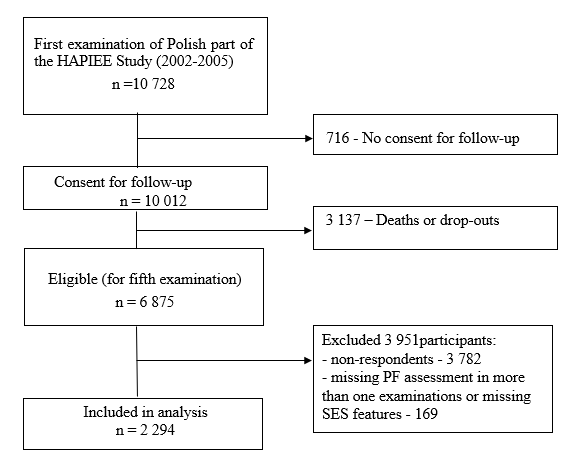


**Supplementary Figure 1**. Study flow diagram.


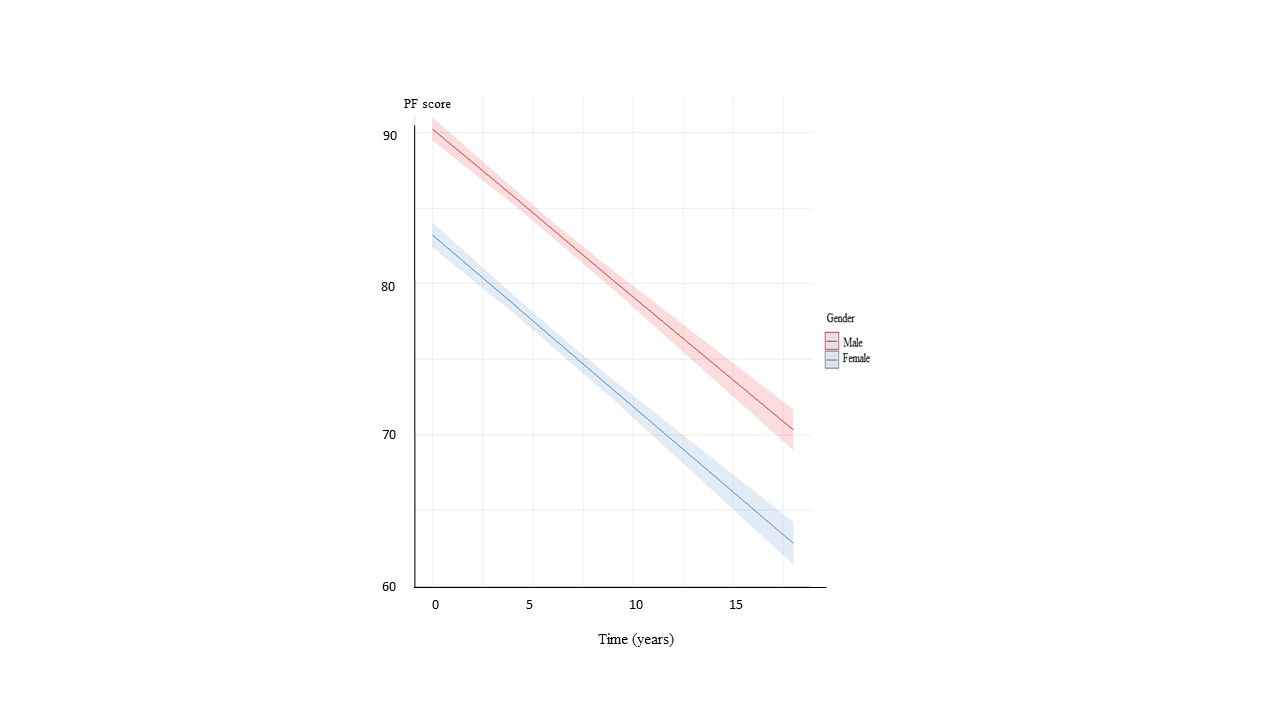


Supplementary Figure 2. Decline in PF in men and in women.
